# Lipid compartments and lipid metabolism as therapeutic targets against coronavirus

**DOI:** 10.3389/fimmu.2023.1268854

**Published:** 2023-12-01

**Authors:** Daniella Cesar-Silva, Filipe S. Pereira-Dutra, Ana Lucia Moraes Giannini, Clarissa M. Maya-Monteiro, Cecília Jacques G. de Almeida

**Affiliations:** ^1^ Laboratory of Immunopharmacology, Department of Genetics, Oswaldo Cruz Institute, Fundação Oswaldo Cruz, Rio de Janeiro, Brazil; ^2^ Laboratory of Functional Genomics and Signal Transduction, Universidade Federal do Rio de Janeiro, Rio de Janeiro, Brazil; ^3^ Laboratory of Endocrinology and Department of Endocrinology and Metabolism, Amsterdam University Medical Centers (UMC), University of Amsterdam, Amsterdam, Netherlands

**Keywords:** lipid nanodomains, caveolae, caveolin, lipid droplets, coronavirus, SARS-CoV-2, lipid metabolism, inflammation

## Abstract

Lipids perform a series of cellular functions, establishing cell and organelles’ boundaries, organizing signaling platforms, and creating compartments where specific reactions occur. Moreover, lipids store energy and act as secondary messengers whose distribution is tightly regulated. Disruption of lipid metabolism is associated with many diseases, including those caused by viruses. In this scenario, lipids can favor virus replication and are not solely used as pathogens’ energy source. In contrast, cells can counteract viruses using lipids as weapons. In this review, we discuss the available data on how coronaviruses profit from cellular lipid compartments and why targeting lipid metabolism may be a powerful strategy to fight these cellular parasites. We also provide a formidable collection of data on the pharmacological approaches targeting lipid metabolism to impair and treat coronavirus infection.

## 1 Introduction

### 1.1 Plasma membrane and cholesterol homeostasis as therapeutic targets in antiviral approaches

Lipids serve multiple functions in the cell. They reside mostly in the cell membranes, where they can be distributed distinctly within membrane leaflets and in cholesterol rich nanodomains according to their biophysical and biochemical properties. These lipid nanodomains, (historically called “lipid rafts”), concentrate coronavirus receptors and their disruption affects the expression and proper localization of coronavirus receptors, impacting virus entry (1–7). Virus structural proteins are also found in these lipid domains due to their affinity to cholesterol and this localization seems to be important for the induction of cell-cell fusion observed during coronavirus infections (6, 8, 9).

At the plasma membrane we can also find caveolae, invaginations with lipid nanodomain-like organization. Although caveolae are not directly relevant for coronaviruses entry, one of its main components, caveolin-1, colocalizes with some coronavirus receptors, seems to be involved in virus infectivity and may participate in virus fusion to the plasma membrane in a cholesterol dependent manner (2, 10).

Besides its role in the membrane, cholesterol participates in coronavirus cycle and host response against virus infection in diverse ways. Key components of the cholesterol metabolism are altered and associated with virus infectivity, inflammatory response, and severity of COVID-19 and MERS (8, 11–13). Cholesterol esterification and triacylglyceride synthesis leads to the biogenesis of lipid droplets, organelles where coronavirus replicate and that contribute to the production of inflammatory mediators (14–18). Cholesterol oxidized products also modulate viral infection and host responses (19–31). Fatty acid metabolism is involved in the acylation of the spike protein, contributing to its localization in lipid nanodomains and trafficking, virus entry and assembly (32–36). Other relevant aspects of lipid metabolism for coronaviruses include the role of specific sphingolipids in virus infection and disease severity (37–45). In this review, we discuss some of these roles and their impact on infectivity and host responses. Besides, we present a large collection of results obtained using *in vitro* and *in vivo* coronavirus infection models that support the therapeutical benefits of using lipid metabolism drugs as strategies against coronaviruses.

We begin with a brief background on lipid domains and cholesterol metabolism to provide the basis for understanding their involvement in coronavirus infection.

Lipid nanodomains are nanoscale cell membrane domains rich in cholesterol, glycosylated, and saturated lipids, such as sphingolipids, assembled in a liquid-ordered (Lo) compact arrangement (46–48). These domains also segregate glycosylphosphatidyl (GPI)-anchored and lipidated proteins, forming signal transduction platforms (47). The existence of lipid nanodomains has been a matter of intense debate in the past, as their visualization can be challenging due to their transient character in quiescent cells. However, upon stimulation, receptors in these domains cluster, inducing lipid nanodomains coalescence, signaling protein recruitment, and interaction with the cortical actin filaments (49, 50). Solubilization of cell membranes with non-ionic detergents at cold temperatures (usually, Triton X-100 at 4°C) allows isolation of domains, segregating GPI-anchored proteins and Lo domain probes. Thus, this method is often used to separate membrane domains representing lipid nanodomains, although there is controversy about this correspondence. More precisely, this approach helps the isolation of Lo domains and the associated molecules, drawing the inference that these molecules would be associated with lipid nanodomains in the living cell (51). Proteins in these domains display specific characteristics that favor their compartmentalization. Moreover, proteins can be anchored in membranes by different lipid modifications by co- or post-translational modifications, some reversible while others are permanent. Such lipid anchors include glycophosphatidylinositol (GPI) anchors, N-terminal myristic acid tails, cysteine acylation, isoprenylation, and the addition of C-terminal sterol moieties. S-acylation is the reversible and enzyme-mediated attachment of a fatty acid to a cysteine residue via a thioester linkage (called palmitoylation because palmitic acid is frequently the added acyl chain). This process targets proteins to the inner leaflet of the cell membrane, preferentially to lipid nanodomains. Cholesterol-depleting and -sequestering drugs, such as MβCD and nystatin disrupt these lipid nanodomains hampering intracellular signaling triggered by molecules organized in these domains.

Caveolae are specialized lipid nanodomains that form invaginations of 50-200 nm in the plasma membrane. These structures likely serve as safeguards when sudden changes in membrane tension occur, for example, during hypotonic shock or mechanical stretch. In these situations, caveolae flatten, providing membranes required for cellular reshaping (52, 53). Caveolae are also crucial for transcytosis (54, 55) and cell signaling. Significantly, they are also involved in controlling lipid metabolism, the main subject of this review (56). Caveolae bear specific proteins, which are determinants for sculpting these structures and executing caveolar functions. These proteins belong to two prominent families: caveolins and cavins. The caveolin family comprises three members of integral membrane proteins named caveolin-1, caveolin-2, and caveolin-3. Caveolin-1 and -2 are ubiquitous but are enriched in endothelial cells, adipocytes, pneumocytes, and fibroblasts, whereas caveolin-3 is specific to muscle cells (57–61). Caveolin-1 and caveolin-3 share a high degree of identity. They are essential for caveolae formation, whereas caveolin-2, the most divergent member of the family, is not required but favors the formation of deeper caveolae (62, 63). The interaction of signaling molecules with caveolins contributes to their compartmentalization and functions. Although the absence of caveolins is not lethal (64–66), their deficiency is associated with various diseases (67–71). The cavin family comprises four cytosolic proteins: cavin1, cavin2, cavin3, and cavin4; the latter being specific to muscle cells. Cavin1 hetero-trimerizes with the other cavins and is essential for caveolae formation (72–74). The other cavins are not essential, except for cavin 2 in endothelial cells of specific tissues (75), but are important to shape and stabilize caveolae (76–79). Lipids play essential roles in caveolae dynamics and functions. Caveolae are enriched in cholesterol, sphingomyelin, and glycosphingolipids, like GD3 (80). Cholesterol binding to caveolin-1 promotes oligomerization (81) and trafficking from the Golgi to the plasma membrane (82). Cholesterol has a vital role in the caveolae structure, which are also disrupted by treatment with cholesterol-depleting drugs (83–85). Furthermore, not only do lipids affect caveolin expression and caveolae formation and motility, but caveolin expression also regulates lipid homeostasis. In the absence of caveolin-1, alterations in cholesterol metabolism occur, such as reduction of free cholesterol synthesis, enhancement of cholesterol esterification (86), and reduction of cholesterol in lipid droplets (87). Caveolin-1 can also be found in lipid droplets where it is believed to play a role in the stabilization, as well as in the lipolysis of these organelles (82, 88–90). Caveolin itself and caveolae as a whole, traffic to lipid droplets, increasing their size and lipid uptake (91). Taken together, all these data corroborate the relevance of caveolae to lipid metabolism.

The lipid droplet (LD) is an organelle where several hydrophobic reactions involved in lipid, energy, and redox homeostasis take place (92–94). Lipid droplets contain a hydrophobic core composed of neutral lipids, mainly triacylglycerol (TG) and cholesterol ester (CE) and are delimited by a monolayer of phospholipids (95–97) and associated proteins (98–100) Although LDs are ubiquitous, their protein and lipid composition strongly depend on the cell type and cellular metabolic state (101–103). Their numbers and sizes are also variable due to the balance between lipid synthesis and degradation (97, 104, 105). The biogenesis of LDs occurs in the ER (106), where critical enzymes involved in lipid synthesis are located, mainly diacylglycerol O-acyltransferases (DGAT1 and DGAT2) (107, 108) and acyl-CoA: cholesterol O-acyltransferases (ACAT1/SOAT1 and ACAT2/SOAT2) (109–111), responsible for the synthesis of TG and CE, respectively (94, 112). The lipid metabolism pathways leading to lipid uptake, cholesterol efflux, autophagy, β-oxidation, lipophagy, and lipid remodeling (109, 113–118) impact LD accumulation or consumption (119–122). Besides, LDs play a role in lipid availability and membrane biosynthesis (123, 124).

Cellular cholesterol content is regulated by uptake, *de novo* synthesis, storage, and export. Diet-derived cholesterol is absorbed by the enterocytes, transported to intracellular organelles, and released in the bloodstream (125), where it circulates associated with lipoproteins, like chylomicrons, very low-density proteins (VLDL), low-density lipoproteins (LDL), and high-density lipoproteins (HDL). LDL is recognized by LDL receptors on cell surface and is endocytosed by clathrin-coated vesicles (126). LDL-bound cholesterol ester (CE) is then hydrolysed by lysosomal acid lipase (LAL) in the lysosomes, from where vesicular and non-vesicular mechanisms transport cholesterol to other organelles (127). The plasma membrane holds 60-90% of this lipid (128). Studies using radiolabeled cholesterol binding probes indicated the existence of three distinct cholesterol pools: one that binds the probe, named the accessible pool, which is sensitive to cholesterol deprivation; the SM-sequestered pool that is not affected by cholesterol deprivation and binds the probe only after SMase treatment; and the essential pool which does not bind to the probe, even after SMase treatment and whose depletion causes rounding and dissociation of the cell from the culture dish. Interestingly, the excess of the accessible pool traffic to the ER, the organelle responsible for cholesterol synthesis, starting with acetyl-CoA and involving more than 20 enzymes (128).

**Figure 1:**
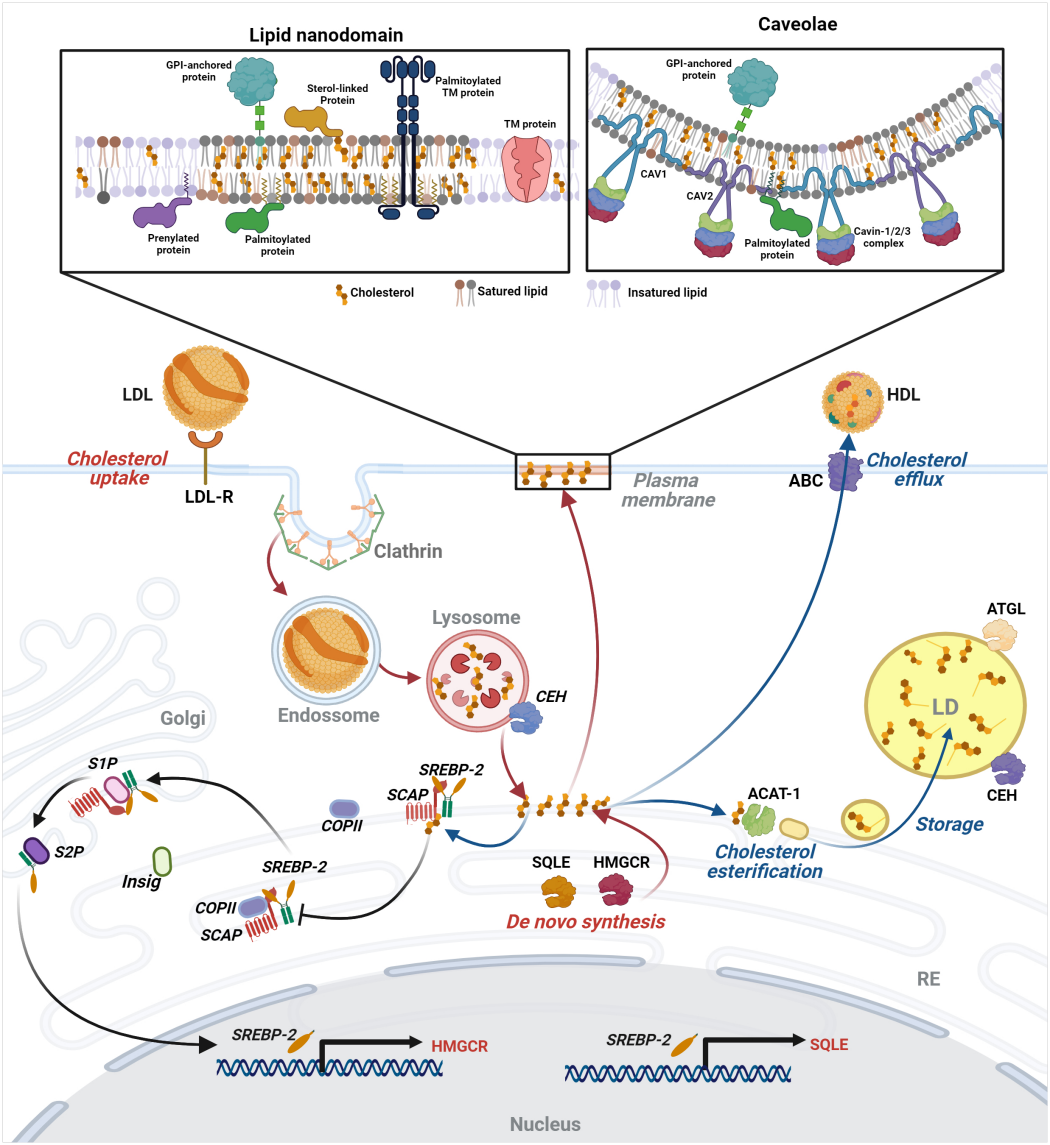
Intracellular lipid compartments and control of cellular cholesterol levels - The cholesterol level in the cell is regulated by uptake, *de novo* synthesis, storage, and export. LDL-bound cholesterol enters the cell by receptor-mediated endocytosis in clathrin-coated vesicles reaching the lysosomes, from which cholesterol is released and transported by vesicular and non-vesicular mechanisms to cellular organelles and the plasma membrane (PM), where it is more enriched in lipid nanodomains and caveolae. For details of these structures, see the text in “Plasma membrane and cholesterol homeostasis as therapeutic targets in antiviral approaches” section. Excess of the accessible cholesterol pool in PM is transported to the ER, where cholesterol synthesis also occurs. There, SREBP2 forms a heterodimer with SCAP, cholesterol level sensor. Low levels of cholesterol result in cleavage and activation of a soluble N-terminus SREBP2 that goes to the nucleus and activates the transcription of genes involved in cholesterol synthesis, such as HMGCR and SQLE. When cholesterol rises in the ER, it binds SCAP, inhibiting the transport of SREBP2 to the Golgi and its processing, halting its activity as a transcription factor. The excess cholesterol can be stored in lipid droplets or exported through ABC-binding cassette (ABC) transporters. Image created using BioRender.

Despite being the center of cholesterol synthesis, the ER disposes of only 1% of the total cellular cholesterol due to the fast transport of newly synthesized cholesterol to cellular membranes or lipid droplets (129). The master regulator of cholesterol synthesis is sterol regulatory element-binding protein 2 (SREBP2), a transcriptional factor synthesized as a precursor protein. In the ER, SREBP2 forms a heterodimer with SCAP (SREBP-cleavage activating protein), a sensor of cholesterol level (130). When ER’s cholesterol level is below 5% of total ER lipids, SCAP binds COPII in transport vesicles and escorts SREBP2 to the Golgi, where it is processed and activated by site 1 (S1P) and site 2 (S2P) proteases (131). The activated soluble N-terminus SREBP2 goes to the nucleus and binds to sterol regulatory elements (SRE) found in genes coding for proteins involved in cholesterol synthesis, like 3-hydroxy-3-methylglutaryl coenzyme A reductase (HMGCR) and squalene monooxygenase (SQLE). When cholesterol levels increase in the ER, cholesterol binds SCAP, blocking its interaction with COPII, thus inhibiting SREBP2 transport to the Golgi. Besides, cholesterol binding to SCAP induces its interaction with INSIG1 and INSIG2 (insulin-induced gene-1 and -2 proteins), which also interferes with SCAP’s association with COPII (132). Furthermore, the cell manages the cholesterol excess by storing or exporting it. Acyl-coenzyme A: cholesterol acyltransferase (ACAT) esterifies cholesterol that can be stored in LDs. CE from lipid droplets is released by the CEHs associated with the lipid droplet surface and is transported to other organelles or the extracellular milieu (133). ABC-binding cassette (ABC) transporters mediate the efflux of non-esterified apolipoprotein-bound cholesterol originated from diverse organelles (**Figure 1**) (134).

In the next sections, we will systematize information about how distinct aspects of lipid metabolism favor coronavirus infection or the host response against it. Moreover, we will present results obtained with a wide variety of drugs that aim the lipid metabolism as a strategy to fight coronavirus infection and the harmful consequences of the triggered host inflammatory response. Finally, we will focus on drugs that were tested in *in vivo* animal models and clinical studies.

## 2 Coronavirus infections subvert the cell lipid metabolism

### 2.1 The first hurdle: the plasma membrane

Coronaviruses bind to specific plasma membrane proteins, for instance, the angiotensin-converting enzyme-2 (ACE2) in the case of SARS-CoV and SARS-CoV-2 and dipeptidyl peptidase 4 (DPP4) in the case of MERS-CoV. Besides these receptors, other molecules facilitate the attachment of these viruses (**Table 1**). After binding to cell receptors, coronaviruses can fuse directly to the plasma membrane or enter by endocytosis mediated by different mechanisms, depending on the virus and the cell type, and that involve lipid nanodomains, clathrin-coated vesicles, caveolae, or clathrin- and caveolae-independent pathways. Direct fusion with the plasma membrane relies on the proteolytic processing of Spike by proteases, like TMPRSS2 in the case of SARS-CoV-2. Otherwise, Spike can be processed by cathepsins activated by the lower pH found in endosomes (146). Thus, the cellular repertoire of receptors, proteases, and other cellular factors affect the virus entry route. For instance, in Calu-3 cells that express TMPRSS2, SARS-CoV-2 enters by fusion, whereas in Vero E6 cells, it enters by an endocytic pathway. The inhibition of infection by drugs that raise the endosomal pH, such as chloroquine, hydroxychloroquine, NH4Cl, and bafilomycin, indicate the requirement of endocytosis for successful infection (147–151).

**Table 1:** Receptors of SARS-COV-2.

Receptors	Class	Gene expression data	References
ACE-2	main receptor	Broad distribution, including human lung epithelial cells	(135, 136)
AXL	alternative receptor	Human lung epithelial cells	(137)
CD147	coreceptor	Human lung epithelial cells	(138)
DC-SIGN	coreceptor	Human IGSF21+ dendritic cells	(139)
GM1	coreceptor	Human cerebrovascular cells	(140)
GRP78	coreceptor	Broad distribution, including human lung epithelial cells	(141)
Heparan sulfate proteoglycans	coreceptor	Broad distribution, including human lung epithelial cells	(142, 143)
L-SIGN	coreceptor	Human macrophages	(139)
NRP1	coreceptor	Broad distribution, including human lung epithelial cells and endothelial cells	(144)
SIGLEC1	coreceptor	Human alveolar macrophages, dendritic cells, and monocytes	(139)
TIM-1	alternative receptor	Human and mouse lung and kidney epithelial cells	(145)

ACE-2 (Angiotensin-converting enzyme 2), AXL (AXL receptor tyrosine kinase), CD147 (cluster of differentiation 147), DC-SIGN (Dendritic cell-specific ICAM-3 grabbing nonintegrin), GM1 (monosialotetrahexosylganglioside), GRP78 (Glucose-regulated protein-78), L-SIGN (Liver/lymph node-specific intercellular adhesion molecule-3-grabbing integrin), NRP1 (neuropilin 1), PBMC (peripheral blood mononuclear cells), SIGLEC1 (Sialic Acid Binding Ig Like Lectin 1), TIM-1 (T-cell immunoglobulin and mucin domain protein 1).

In several studies, lipid nanodomains were implicated in coronavirus entry. Disruption of lipid nanodomains by cholesterol-depleting drugs, like MβCD, reduces coronavirus infectivity, whereas cholesterol replenishment restores virus infectivity (1–6). Nevertheless, the timing is crucial for this inhibition as MβCD pretreatment impairs SARS-CoV infection in Vero E6 cells, whereas a 3h post-treatment has no effect, indicating that cholesterol depletion hampers early steps of the virus cycle (3). Similarly, pretreatment, but not post-treatment with simvastatin, a cholesterol-reducing drug, inhibits SARS-CoV-2 replication in Vero E6 and Calu-3 cells. Accordingly, simvastatin impairs binding and entry of SARS-CoV-2 in Calu-3, possibly due to the dislodgment of ACE2 from lipid nanodomains caused by the drug and despite the increase in ACE2 expression that both simvastatin and infection provoke in these cells (7). MβCD treatment also induces relocation of ACE2 from nanodomains to membrane non-raft regions without impacting ACE2 expression levels in Vero E6 or Caco-2 cells (4, 5). Furthermore, a tagged-ectodomain of the SARS-CoV Spike also colocalizes with detergent-resistant membrane domains and with GM1 in fixed cells, which indicates the presence of ACE2 in lipid nanodomains upon Spike binding (4). In contrast to the view that the presence of ACE2 in lipid nanodomains favors SARS-CoV-2 infection, Wing and coworkers (2023) showed that avasimibe - an ACAT inhibitor - increases both free cholesterol in the plasma membrane and the size of GM1-enriched lipid nanodomains, as well as the ACE2 localization in these domains, while reducing SARS-CoV-2 attachment to Vero E6 cells (152) Structural proteins of the avian infectious bronchitis virus (IBV) also localize to detergent-resistant fractions and move to soluble fractions after MβCD treatment. MβCD, as well as mevastatin, another member of the statins’ drug class, also reduces the expression of these proteins (6). In contrast, Li and coworkers (2007) could not detect ACE2 in lipid nanodomains using Vero E6 cells and reported decreased ACE2 expression after MβCD treatment (3). This cholesterol-depleting drug also inhibits mouse hepatitis virus (MHV) replication. Still, the mechanisms cannot be attributed to the relocation of its receptor since CEACAM is detected in detergent-resistant membrane domains when a mild concentration of cold Triton X-100 (0.2%) is used to isolate this receptor in CEACAM-expressing HeLa cells membranes. However, an exogenous GPI-anchored CEACAM expression in detergent-resistant domains (isolated with 1% Triton X-100) fails to enhance MHV infection (1). Nevertheless, Choi and coworkers demonstrated that in the presence of MHV, CEACAM, and Spike redistribute into lipid nanodomains in CEACAM-overexpressing HEK293 cells. These data suggest that cholesterol depletion by MβCD does not affect virus binding but reduces virus entry drastically, possibly because viral proteins redistribute and interact with lipid nanodomains. Spike is detected only in detergent-soluble membranes of the Golgi, meaning that Spike is not associated with lipid nanodomains during virus assembly and budding but is associated with detergent-resistant membrane domains at the plasma membrane, which is important for Spike-induced cell-cell fusion (153). Accordingly, Spike of coronaviruses contains several cholesterol-binding motifs in their sequences (8, 9).

Cholesterol also participates in distinct entry pathways, affecting caveolae- and clathrin-mediated endocytic mechanisms (149, 154). Therefore, excluding alternative entry routes using specific tools is greatly valuable. Wang and coworkers report lipid raft-mediated entry of SARS-CoV in a mechanism independent of caveolae and clathrin in Vero E6 and HEK293-ACE2-GFP cell lines. Clathrin-mediated endocytosis was excluded based on experiments using chlorpromazine (an inhibitor of clathrin-mediated endocytosis), by silencing the clathrin coding gene, or by expressing a dominant-negative Eps15, which participates in clathrin-mediated endocytosis. Caveolae-dependent endocytosis was also excluded by experiments using filipin or nystatin, which inhibited endocytosis of cholera toxin B, used as a control but failed to inhibit SARS-CoV endocytosis. Moreover, caveolin-1 does not colocalize with the SARS-CoV Spike (155).

On the other hand, other studies found that SARS-CoV and SARS-CoV-2 invade HepG2 cells (a hepatocarcinoma cell line) and HEK-293-ACE2 cells, respectively, by clathrin-mediated endocytosis (156, 157). MHV-2 also uses clathrin-mediated endocytosis to enter the mouse astrocytoma DBT cells, which was demonstrated by using chlorpromazine, hypertonic sucrose medium, or by silencing the clathrin heavy-chain gene. Notwithstanding, the clathrin-mediated endocytosis of MHV-2 is Eps15-independent. Besides, the overexpression of a dominant-negative mutant of caveolin-1 did not affect the invasion of MHV-2 in DBT cells, ruling out the participation of this protein or caveolae in virus entry (158). Porcine hemagglutinating encephalomyelitis virus (PHEV) also uses clathrin-mediated endocytosis to invade mouse neuroblastoma cells (Neuro-2a). Entry by endocytosis is evidenced by virus presence in coated pits and EEA1-decorated vesicles and by the inhibition of invasion induced by pH-raising agents, such as chloroquine and NH4Cl. Clathrin’s involvement in PHEV entrance is further demonstrated by PHEV and transferrin coincidental labeling, chlorpromazine sensitivity, and low infectivity detected in a dominant-negative clathrin mutant-expressing or a clathrin heavy chain-silenced cell line. Additionally, silencing of caveolin-1 does not affect PHEV entry in Neuro-2a cells (150).

Other studies propose that caveolin-1, and not necessarily caveolae, are involved in coronavirus infection. The human coronavirus 229E binds to CD13, enriched in membrane detergent-resistant domains where it colocalizes with caveolin-1. MβCD does not hamper virus binding to the cell surface but reduces the colocalization of 229E with caveolin and virus infectivity, while cholesterol replenishment reverses these effects. The silencing of caveolin-1 does not impair 229E binding but reduces its entrance into the cell significantly. 229E particles are detected close to caveolae, but interestingly they are not found in the lumen of caveolae or endosomes. Thus, caveolin-1 may participate in the fusion of the virus particle with the plasma membrane but not in caveolae-mediated endocytosis of the 229E virus (2). Notably, epithelial cells, the primary targets for coronaviruses, do not present caveolae in their apical surfaces, although they express caveolin-1 on these sites (159, 160). The human coronavirus OC43, which uses HLA class I molecule or sialic acid as receptors, colocalizes to caveolin-1 in HCT-8 cells during the early phase of infection. Nystatin, MβCD, or *CAV1* silencing inhibits virus invasion and infectivity. The requirement of dynamin was also proved using dynasore and MiTMAB (10). Zhou and colleagues (2022) further showed that SARS-CoV-2 pseudovirus uses CD147 to enter Vero E6 and Huh-7 cells and exploit an Arf-6-dependent caveolar/lipid raft pathway (161).

Enveloped viruses may also present cholesterol in their membranes (162, 163), and cholesterol depletion from the viral envelope of the transmissible gastroenteritis virus (TGEV) by MβCD reduces its infectivity drastically, whereas cholesterol replenishment reverts this effect (164). Curiously, the Spike protein, found in detergent-resistant domains of the TGEV envelope membrane, does not become detergent-soluble after MβCD treatment and does not segregate with the lipid raft marker flotillin-2, indicating that the viral membrane organization is different from that of the plasma membrane (165).

### 2.2 Coronavirus infection reshapes sphingolipids metabolism

Structural and modeling studies identified that the N-terminal domain of the Spike protein of SARS-CoV-2 interacts with ganglioside sialic acids (166) (see **Table 1**). SARS-CoV-2 and other betacoronaviruses use these molecules to attach and invade host cells (140, 167). Interestingly, gangliosides are a group of sphingolipids enriched in lipid nanodomains.

**Figure 2:**
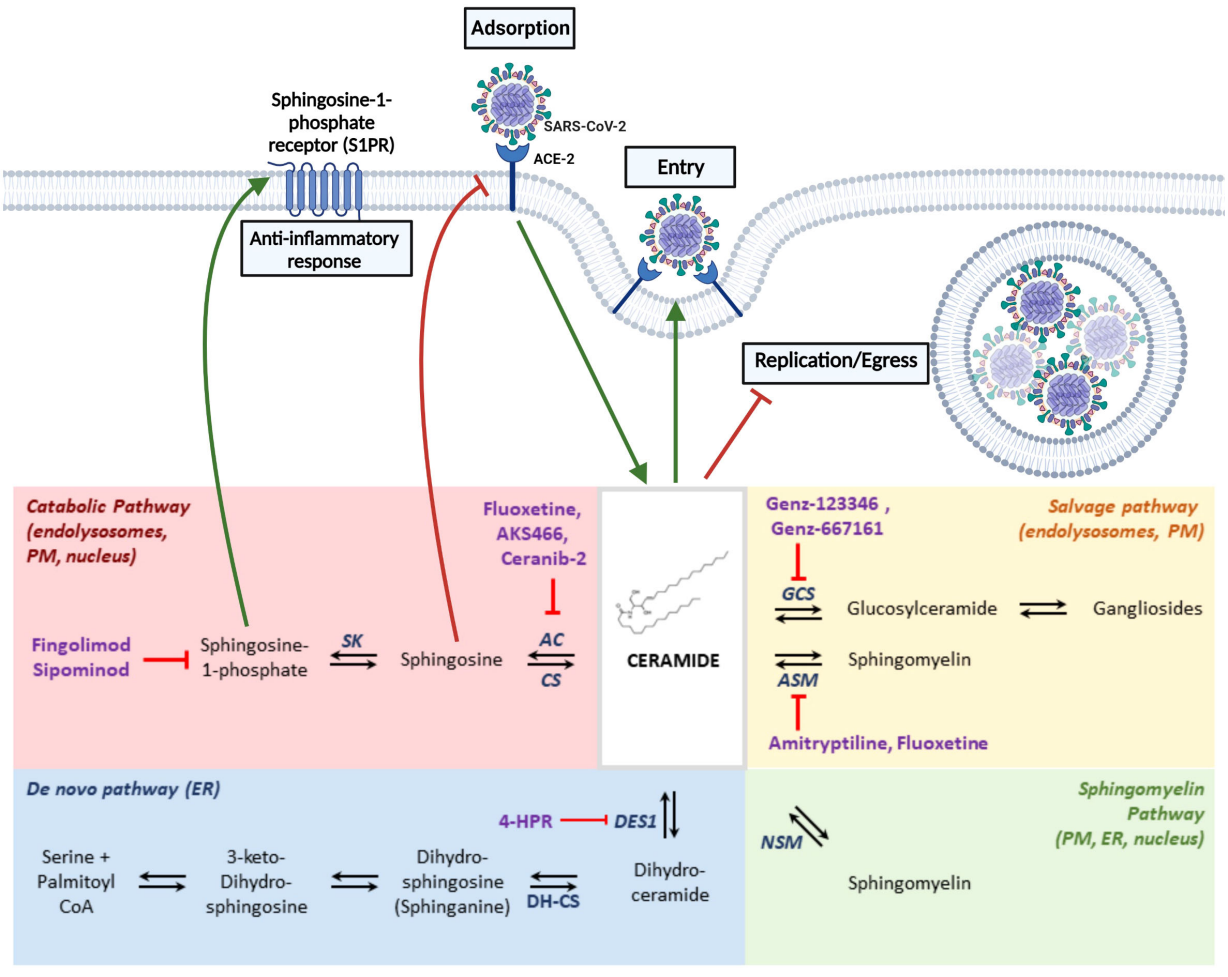
Coronaviruses infection and sphingolipid metabolism -The central hub of sphingolipid metabolism is ceramide, which can be synthesized by hydrolysis of sphingomyelin, by *de novo* synthesis, or by recycling from complex sphingolipids. Ceramide has a dual role during SARS-CoV-2 infection, promoting viral entry when it accumulates in the plasma membrane or blocking viral egress of viral particles that accumulate in the endolysosomes. The interaction of ACE2 and Spike activates acid sphingomyelinase (ASM) that metabolizes sphingosine into ceramide, which favors SARS-CoV-2 infection. Inhibition of ASM by various drugs suppresses SARS-CoV-2 infection. Inhibition of acid ceramidase (AC) that metabolizes ceramide into sphingosine also inhibits SARS-CoV-2 infection, as it reduces ceramide levels and increases sphingosine levels that hamper the interaction of Spike with ACE2. Furthermore, sphingosine is metabolized into sphingosine-1-phosphate, which activates an anti-inflammatory response. Drugs inhibiting the activity of enzymes of the sphingolipid metabolism interfering with coronavirus infection are depicted in this figure. AC, acid ceramidase; ASM, acid sphingomyelinase; CS, ceramide synthase; DES1, dihydroceramide D4-desaturase 1; DH-CS, dihydroceramide synthase; GCS, Glucosylceramide Synthase; NSM, neutral sphingomyelinase; SK, sphingosine kinases. Image created using BioRender.

In addition to functioning as alternative receptors, sphingolipids metabolism is involved in other aspects of SARS-CoV-2 infection. The central hub of sphingolipid metabolism is ceramide, which can be synthesized by sphingomyelin hydrolysis, *de novo* synthesis, or recycling from complex sphingolipids (**Figure 2**). COVID-19 patients’ sera display increased levels of dihydrosphingosine, dihydroceramides, ceramides, sphingosine, and acid sphingomyelinase (ASM) correlating positively with disease severity. In contrast, the level of sphingosine-1-phosphate (S1P) correlates negatively with the disease severity (37). Accordingly, S1P can activate the S1P receptor and induce anti-inflammatory responses. Another study shows that patients with symptomatic COVID-19 have diminished serum sphingosine levels compared to asymptomatic counterparts, who also exhibit elevated acid ceramidase (AC), an enzyme that converts ceramide in sphingosine (38). Accordingly, *in vitro* treatment of Vero or human primary epithelial cells with exogenous sphingosine protected these cells from infection by pseudoviruses expressing the SARS-CoV-2 Spike protein, possibly due to the binding of the exogenous sphingosine to ACE2, hampering Spike’s interaction with its receptor (39). Notably, in this pseudovirus model, the interaction of ACE2 and Spike activates ASM. This enzyme metabolizes sphingosine into ceramide in acidic endolysosomes and at the plasma membrane at the physiological pH after being released by endolysosomes (168). In the plasma membrane, ASM is important for lipid raft formation in the outer leaflet. In contrast, neutral SM generates ceramide in the inner leaflet, where it interacts with intracellular signaling molecules (169). Increased ceramide in the outer leaflet of the plasma membrane favors SARS-CoV-2 infection. Accordingly, inhibition of ASM by various antidepressant drugs (either selective serotonin reuptake inhibitors (SSRI) or tricyclic class) or silencing of the *ASM* gene suppresses SARS-CoV-2 infection in Caco-2 cells. Importantly, nasal epithelial cells isolated from amitriptyline-treated (tricyclic class) volunteers were protected from SARS-CoV-2 infection (40). Fluoxetine, an SSRI, also suppresses the replication of different SARS-CoV-2 variants of concern in Vero and A549-hACE2-TMPRSS2 cells, human lung slices (170), and human airway organoid epithelia (41). Geiger and coworkers (2022) showed that fluoxetine and AKS466 - its ASM-independent derivative - target acid ceramidase, leading to the retention of SARS-CoV-2 in lysosomes and inhibiting virus replication in Huh.7, Vero and Calu-3 cells. Ceranib-2, a specific inhibitor of AC, also blocks SARS-CoV-2 replication (42) Further, fluoxetine treatment reduces IL-6 and NF-κB signaling in various cell lines (171). Gene silencing of glucosylceramide synthase (converts ceramide to glucosylceramide), and inhibition by Genz-123346 or Genz-667161, block SARS-CoV-2 early replication steps (43). The same group showed that SARS-CoV-2 infection raises the levels of glycosphingolipids, like dihydrosphingosine, sphingosine, GA1, and GM3, an effect reversed by Genz-123346. This inhibitor does not significantly alter the levels of ceramide, lactosyl ceramide, SM, and GM2 (44). 4-HPR, an inhibitor of dihydroceramide D4-desaturase 1 (DES1- catalyses dihydroceramide in ceramide), inhibits cell-cell fusion and SARS-CoV-2 infection. However, the addition of ceramide fails to revert the effects of 4-HPR. DES1 knockout cells exhibit a similar cell-cell fusion efficiency, despite the enhanced amounts of dihydrosphingosine-derived lipids compared to total sphingolipids. Therefore, the inhibitory effect of 4-HPR on SARS-CoV-2 infection does not rely on DES1, and it is also not related to ACE2 expression or localization. Still, it can be related to the drug effects on decreased membrane fluidity (45).

In conclusion, the metabolism of sphingolipids can determine the outcome of SARS-CoV-2 infection. Ceramide has a dual role in SARS-CoV-2 infection, promoting viral entry when it accumulates in the plasma membrane (40) and blocking viral egress of the viral particles that accumulate in the endolysosomes (42). The balance of ceramide and sphingosine seems crucial during the entry stage since engagement of Spike and ACE2 induces the conversion of sphingomyelin to ceramide favoring infection, whereas the conversion of ceramide to sphingosine blocks ACE2 and Spike’s interaction (39, 40). Patients’ shingolipidome shows that the severity of COVID-19 correlates to ceramide accumulation and S1P reduction (37, 38). S1P results from sphingosine phosphorylation and has anti-inflammatory effects, modulating endothelial cell chemotaxis and barrier integrity. While ceramide and sphingosine promote apoptosis, cell cycle arrest, and differentiation, S1P promotes proliferation and survival (172). Thus, S1P reduction may contribute to the exacerbated inflammation observed in severe COVID-19. For this reason, the S1P modulators, fingolimod, and siponimod, used to treat multiple sclerosis, have been proposed as therapy for treating COVID-19 patients (**Figure 2**) (173, 174).

### 2.3 25-oxysterol antiviral effects

Beyond the essential role of cholesterol in structuring lipid nanodomains and involvement in coronavirus entry, its metabolism affects coronavirus infection and host defense mechanisms. Viral infection activates antiviral responses in the host cell, including the transcription of interferon-induced genes (ISGs), such as the cholesterol 25-hydroxylase (*CH25H*) gene, whose product modifies cholesterol into 25-hydrocholesterol (25HC). 25HC has several antiviral and anti-inflammatory activities in infection by diverse enveloped viruses, including coronaviruses. SARS-CoV-2 infection stimulates the expression of *CH25H* in Calu-3 and A549-ACE2 cells. The overexpression of *CH25H* or the treatment with exogenous 25HC abolishes infection by SARS-CoV, MERS-CoV, or SARS-CoV-2 pseudoviruses in Calu-3 cells. Mechanistically, 25HC induces cholesterol trafficking from the plasma membrane to the endoplasmic reticulum, where the ACAT esterifies it, resulting in the formation of lipid droplets and cholesterol depletion in the plasma membrane, restricting the entry of the virus by fusion with the cell surface (19). Heissler and collaborators (2023) report that 25-HC inhibits the common cold coronavirus hCoV-OC43 infection of CHO-K1 cells. They showed that 25HC suppresses SREBP-2 activation, inhibiting cholesterol biosynthesis and activation of ACAT. The latter results in cholesterol sinking into lipid droplets and depletion of the accessible cholesterol from the plasma membrane, which was confirmed by using anthrolysin O (ALOD 4) that binds specifically to this cholesterol pool. Interestingly, 25HC does not alter SM-sequestered cholesterol (20), indicating that 25HC acts independently of lipid nanodomains. In rabbit blood cells that are devoid of internal membranes, the authors show that 25HC is no longer able to induce a reduction of cholesterol at the plasma membrane, suggesting that 25HC effects are dependent on cholesterol transport from the plasma membrane to other organelles (20). Another study indicates that porcine epidemic diarrhea virus (PEDV) downregulates *CH25H* expression in Vero cells. However, the overexpression of *CH25H* or 25HC treatment also blocks PEDV infection. Interestingly, CH25H-M, a CH25H mutant lacking hydrolase activity and not producing 25HC, also inhibits PEDV infection, although to a lesser extent. CH25H affects the entry of PEDV and the replication of another porcine coronavirus, the TGEV (21). The infection of IPI-FX cells with porcine deltacoronavirus (PDCoV) upregulates *CH25H* expression, and 25HC treatment impairs PDCoV infection, not at the adsorption nor at the replication steps (22), but possibly post-entry steps of PDCoV infection such as replication or egress, at least using LLC PK1 cells (23). PDCoV infection induces a transitory increase in IFN-γ and the production of lipid droplets, while 25HC enhances both effects. Furthermore, infection activates the TGF-β signaling pathway, which 25HC reverses. Asiaticoside, an inhibitor of TGF-β, also inhibits PDCoV infection (23). The overexpression of *CH25H* or 25HC treatment also impairs the infection of HEK293-hACE2 cells by SARS-CoV or SARS-CoV-2 pseudoviruses. The authors show that 25HC accumulates in late endosomes and lysosomes and impairs Spike-mediated membrane fusion (24). The antiviral activity of 25HC was also demonstrated by Yuan and collaborators using the SARS-CoV-2 N antigen expression, viral load reduction, and plaque reduction assays in Vero E6 cells (25). Another relevant aspect of 25HC action on coronavirus infection is the modulation of the inflammatory response since severe cases are characterized by an exacerbated pro-inflammatory response that potentially progresses to organ failure and death. 25HC provides a negative feedback loop of SREBP-dependent activation of pro-inflammatory cytokines (26). Plasma samples of severe COVID-19 patients compared to mild cases show decreased levels of 25HC and increased cytokine levels that correlate with the disease’s severity. Accordingly, *CH25H* expression is also reduced at mRNA and protein levels in peripheral blood mononuclear cells (PBMC) of severe COVID-19 patients. In this context, treatment of PBMC with 25HC inhibits SREBP activity, the expression of pro-inflammatory genes, such as TNF-α, IL-1β, NOX2, and NLPR3, and the production of inflammatory cytokines, such as IL-6, TNF-α, IL-1b, IFN-γ, IL-8, CCL2, among others (27). In contrast, Zu and collaborators (2020) described a sudden increase of 25HC in the plasma of a severe COVID-19 patient two days before her death, as well as an increase in 25HC levels in serum samples of SARS-CoV-2-infected hACE2 mice compared to the control group. Caco-2 cells also exhibit *CH25H* upregulation upon SARS-CoV-2 infection, and the overexpression of *CH25H* inhibits SARS-CoV-2, as well as SARS-CoV-2 pseudovirus particle production in Vero cells, indicating an effect at the entry step of viral infection. 25HC also protects mice from infection by SARS-CoV-2 strain MASCp6.10 (28). Despite the controversial results regarding 25HC levels in the blood upon SARS-CoV-2 infection, both studies reveal a protective role of 25HC against either viral infection or the excessive production of inflammatory mediators caused by infection. In sharp contrast with all these papers reporting a protective role of 25HC, Fessler and collaborators (2022) show that 25HC is effective in controlling the infection caused by the common cold coronavirus (hCoV)-229E, but not the infection and associated damage inflicted by the SARS-CoV-2 (29). Regarding 229E infection, 25HC reduces viral plaque number and size in MRC-5 cells, indicating a post-entry effect. On the contrary, 25HC does not affect SARS-CoV-2 infection in TMPRSS2-expressing VeroE6 cells. Likewise, despite the increase of *CH25H* expression in K18-hACE2 mice, 25HC treatment does not change the viral load in these mice. Corroborating to the inefficacy of 25HC, *CH25H*-deficient mice show a similar viral load to wild-type mice. Furthermore, 25HC does not change inflammatory lung indicators to SARS-CoV-2 infection, such as the level of inflammatory mediators and total cell counts in bronchoalveolar lavage fluid (BALF). Of note, 25HC increases inflammatory chemokines in the plasma of infected treated mice and microvascular leakage, and it does not improve lung damage or the survival rate. These observations recommend caution regarding the therapeutic potential of 25HC (29). It is worth mentioning that Marcello and coworkers failed to find any correlation between 25HC in the blood of COVID-19 patients and disease severity. Instead, they observed a 50% decrease in 27HC in COVID-19 patients. *In vitro*, 27OH inhibits infection of SARS-CoV-2 and HCoV-OC43 (30). The role of other oxysterols in COVID-19 and other coronavirus pathologies still deserves more studies, such as the one by Ohashi and coworkers (2021) describing anti-SARS-CoV-2 activities (31). Finally, we would like to mention that 25HC inhibits caveolin-1 expression and the subsequent Cav-1+-endossome-dependent IFN-γ signaling in the context of Parkinson’s disease (175), an aspect of the regulation of the inflammatory response not investigated so far in coronavirus infection.

### 2.4 Coronavirus infection reshapes cholesterol metabolism

Wei and collaborators (2020) correlated SARS-CoV-2 infection with lipoprotein metabolism. First, they demonstrated that SARS-CoV-2 Spike can bind cholesterol and that HDL increases the entry of a pseudovirus expressing SARS-CoV-2 Spike-in Huh-7 cells in a dose-dependent manner. Furthermore, HDL also increases the entry and replication of SARS-CoV-2, a process dependent on the scavenger receptor B type 1 (SR-B1). ACE2 and SR-B1 are co-expressed in pulmonary and extrapulmonary tissue such as the retina, colon, and liver, indicating that SR-B1 facilitates SARS-CoV-2 infection and explaining the viral tropism for these organs (8).

The total cholesterol, HDL, and LDL levels in the blood of COVID-19 patients correlate negatively with infection and disease severity (11). On the other hand, SREBP and NFκB activation are higher in fatal cases compared to survivors. Moreover, the C-terminal fragment of SREBP-2 is present in the blood of COVID-19 patients and correlates to disease severity, cytokine levels, and vascular damage, being higher in septic patients. Treatment of PBMC from COVID-19 patients with fatostatin A (an SREBP-2 processing inhibitor) and SN50 (an NF-κB signaling inhibitor) suppress the production of pro-inflammatory TNF-α and IL-1β. Accordingly, the prototypical pro-inflammatory lipopolysaccharide (LPS) stimulation of HUVEC cells provokes an increase in the C-terminal SREBP content in cell culture lysates and supernatants, whereas the N-terminal SREBP increases only in the cell lysates. These results indicate that the liberation of SREBP C-terminal fragment correlates to inflammatory stimulation and may be a useful indicator of disease severity in COVID-19 patients and a possible target to reduce the exacerbated inflammation response in severe COVID-19 (12). Besides, the same study showed that *SREBP-2* silencing blocks the LPS effect. Fatostatin, SN50, and *SREBP-2* silencing all diminish sepsis outcomes of the cecal ligation and puncture (CLP) model of sepsis. The authors propose that SARS-CoV-2 infection leads to C-terminal SREBP release, which induces cytokine storm production and the vascular damage observed in COVID-19 septic patients (12). Pharmacological intervention on the transactivation of lipogenic enzymes by the N-terminal SREBP also suppresses MERS-CoV infection, whose primary target is the lipid metabolism pathway, at least in the Calu-3 cells infection model. Using a lipid metabolite library, Yuan and coworkers (2019) identified that AM580, a synthetic analog of retinoic acid receptor alpha (RARα), was protective against MERS-CoV infection in the hepatic cell line Huh7 (13). RARα regulates cholesterol metabolism by stimulating the expression of Apo-A1 in hepatic cells (176), as well as of cholesterol efflux transporters (ABCA1 and ABCG1) in macrophages (177). MERS-CoV infection triggers the expression of genes coding lipogenic enzymes like acetyl-CoA carboxylase (ACC), fatty acid synthase (FAS), and hydroxymethylglutaryl-CoA synthase (HMGCS), besides enhancing the production of lipid droplets. Not surprisingly, AM580 suppresses these effects and reduces MERS-CoV replication drastically in various cell lines, such as Calu-3, A549, Vero, THP-1, human primary monocyte-derived macrophages (MDMs), human primary small airway epithelial cells (HSAEC), as well in human intestinal organoids. AM580 binds to the N-terminus forms of SREBP1 and SREBP2, preventing lipogenic genes’ transactivation. The effect of AM580 is counteracted by exogenous palmitate, indicating that MERS-CoV subverts the fatty acid metabolism to its benefit. AM580 also abolishes the inflammatory response in Huh7 cells and MDMs (13). AM580 is further effective *in vivo*, as described in session 5 of this review. In the following sections of this review, we will contextualize other aspects of coronaviruses’ subversion of cholesterol metabolism.

### 2.5 Coronavirus infection remodels fatty-acid metabolism

#### 2.5.1 Fatty acid *de novo* synthesis during coronavirus infection

Drugs addressed to fatty acid metabolism are also effective against coronavirus infection. Orlistat, a drug that inhibits gastric lipases and FAS, and triacsin C, an inhibitor of long-chain acyl CoA synthetase (ACS), both inhibit SARS-CoV-2 infection in VeroE6 cells when added 2h or 4h post-infection, but have minimal effect if cells are treated for 1h before infection, indicating a post-entry action. Orlistat, triacsin C, and other inhibitors that impair *de novo* fatty acid synthesis, such as TOFA (an ACC inhibitor) and C75 (a FAS inhibitor), also block SARS-CoV-2 infection in Calu-3 cells (14).

Besides their essential role in phospholipids syntheses, fatty acids participate in protein lipidation, energy generation, and lipid droplet formation. The inhibition of β−oxidation by etomoxir (an inhibitor of carnitine palmitoyl-transferase 1A -CPT1A) and trimetazidine (an inhibitor of beta-oxidation of long-chain 3-keoacyl-CoA thiolase) do not impact SARS-CoV-2 replication. On the other hand, the inhibition of palmitoyl acyltransferase (PAT) by 2-bromopalmitate hinders SARS-CoV-2 infection. Accordingly, *FASN*-deficient cells do not sustain SARS-CoV-2 replication. In contrast, incubation with palmitic acid and oleic acid supports replication in these cells, further demonstrating the central role of fatty acid metabolism during SARS-CoV-2 infection (14). Chu and coworkers (2021) demonstrate the importance of palmitoylation for SARS-CoV-2 using an experimental setting of genome silencing in HEK293T-hACE2 and Caco-2 cells with a library of metabolic shRNAs, followed by infecting these cells with an infectious-clone-derived SARS-CoV-2 carrying a mNeoGreen reporter protein (SARS-CoV-2-mNG virus). This study identified host factors that favor virus replication, such as *FASN* (which encodes FAS) and *ACACA* (which encodes ACC1). *FASN*-KO HEK293T-hACE2 cells are also resistant to virus infection, and eleven out of 22 FAS inhibitors blocked SARS-CoV-2-mNG infection, the most effective ones being TVB-3664, orlistat, TVB-2640, TVB-3166, GSK-214069, FASN-IN-4, and FT113. The first three were effective against the early lineage of SARS-CoV-2 (USA_WA1/2020) and the alpha, beta, and delta variants. BSA-conjugated palmitic acid reverses the inhibition mediated by these inhibitors, indicating that their effect relies on hampering palmitoylation. The orlistat effect is not fully reversed, possibly because this drug also inhibits lipases (178). According to the role of fatty acid metabolism in virus infection, the *FASN* gene is an interferon-regulated gene suppressed by IFN type I after infection by several viruses, including SARS-CoV-2. The artificial overexpression of *FASN* increases SARS-CoV-2 load in HeLa-ACE2 and Huh 7.5 cells, whereas knockdown of *FASN* decreases SARS-CoV-2 replication in HEK293-ACE2 and Huh 7.5 cells. The *FASN* inhibitors C75, epigallocatechin gallate (EGCG), Cerulenin, and TVB-3166 were also effective in inhibiting SARS-CoV-2 infection in HeLa-ACE2, Huh 7.5 cells, and VeroE6 cells (179). Interestingly, Tang and coworkers (2023) identified that SARS-CoV-2 infection in organoids is regulated by the circadian-associated repressor of transcription (CIART) gene, which regulates fatty acid metabolism by activating the NR4A1 (180).

#### 2.5.2 Acylation of the Spike protein

Palmitoylation is vital for coronavirus infectivity, at least in part because of the S-acylation of Spike (32, 33). This modification has several roles that different studies have demonstrated. For instance, palmitoylation protects SARS-CoV Spike from degradation and confers the ability to partition in detergent-resistant membranes (32, 34, 35). However, others reported that SARS-CoV-2 Spike palmitoylation is unnecessary for its location on lipid nanodomains (33). Palmitoylation of SARS-CoV-2 Spike is also crucial for trafficking to the Golgi and the plasma membrane (36). This section will describe what has been published regarding palmitoylation and coronavirus infection.

Spike proteins from several coronaviruses are heavily palmitoylated in cysteine residues, e.g., SARS-CoV-2 Spike is palmitoylated in 10 cytosolic residues (32, 181–183). Besides, Spike protein with cysteine-to-alanine mutations cannot be palmitoylated, and pseudoviruses bearing these mutated versions of Spike fail to enter cells due to hindered fusion of virus envelope and the plasma membrane (33, 181, 182). Interestingly, the mutation of specific cysteines impacts infectivity at different rates (184).

Palmitoylation is mediated by palmitoyl-transferases (PAT), a family of 23 proteins containing a DHHC motif. Although most PAT proteins reside in the Golgi, ZDHHC5 is enriched in the plasma membrane and somewhat in the endosomal system (185). ZDHHC5 and its accessory protein Golgin A7 (GOLGA7) interact with the SARS-CoV-2 Spike protein (33, 186). Accordingly, ZDHHC5 knockdown impaired SARS-CoV-2 Spike palmitoylation and reduced Spike-mediated cell-cell fusion and pseudovirus entry in HEK293T cells (33). In contrast, Zeng and coworkers (2021) showed that ZDHHC5 and GOLGA7 knockout do not affect SARS-CoV-2 Spike palmitoylation nor its localization but somehow impaired virus entry (187). These results could be explained by the fact that other ZDHHCs also bind SARS-CoV-2 Spike (182). Mesquita and coworkers (2021) claim that ZDHHC20 is the main acetyltransferase that lipidates the SARS-CoV-2 Spike. They knocked down each of the 23 ZDHHC acetyltransferases and reported that silencing of ZDHHC8, ZDHHC9, and ZDHHC20 reduced palmitoylation of ectopically expressed Spike. In their study, ZDHHC5 knockdown did not affect Spike palmitoylation (35). Besides acylation with palmitate, coronavirus Spike can also be modified by myristate and stearate, and inhibition of acylation by the FASN inhibitor TVB3166 reduces 229E spread (188). C75 and 2-bromopalmitate (2-BP), inhibitors of ZDHHCs, decrease the palmitoylation of MHV and SARS-CoV-2 Spike and inhibit the entry of SARS-CoV-2 Spike pseudotyped virus (32, 33). Ramadan and coworkers (2022) used bis-piperazine derivatives, which are more specific palmitoylation inhibitors, and reported inhibition of SARS-CoV-2 infectivity (189).

Besides the role in Spike palmitoylation, silencing of ZDHHCs reduced by 30% the amount of cholesterol in virions, which might be related to reduced virus entry. Furthermore, Mesquita and coworkers (2021) showed that palmitoylation of Spike is responsible for the modification of the lipid environment surrounding it, promoting the formation of detergent-resistant domains both in the virion envelope and in the ERGIC, being crucial for the fusion of SARS-CoV-2 pseudoparticles and viral-like particles with the host cell (35). This observation is interesting since the ERGIC usually has a low cholesterol content. Indeed, in uninfected cells, ERGIC53 (a marker of ERGIC) is detergent-soluble but becomes insoluble after SARS-CoV-2 infection. Moreover, the interaction of the MHV Spike with the M protein and their subsequent incorporation into virions depend on Spike palmitoylation (32). This effect is specific to each coronavirus since palmitoylation is dispensable in the case of SARS-CoV and TGEV Spike proteins (34, 183).

In conclusion, Spike acylation assures coronavirus particles’ entry, assembly, and spreading.

#### 2.5.3 Coronavirus infection rewires lipid droplets metabolism

Lipid droplet formation has been detailed in a previous section. Here we will describe the results of several groups that link lipid droplets and coronavirus infections. As mentioned before, fatty acids are used to produce neutral triacylglycerides packaged into lipid droplets. Intriguingly, a consequence of SARS-CoV-2 infection is the accumulation of lipid bodies in monocytes isolated from COVID-19 patients and in SARS-CoV-2 *in vitro* infected primary monocytes from healthy donors. The infection results in the expression of genes coding proteins involved in lipid uptake and lipid metabolic pathways, like CD36, PPARγ, SREBP-1, and DGAT-1 (15). Accordingly, inhibiting DGAT - involved in lipid droplet formation - with A922500 and Xanthohumol reduces SARS-CoV-2 replication. These treatments also inhibit the production of pro-inflammatory mediators by monocytes. A922500 was also effective in restraining SARS-CoV-2 infection on VeroE6 cells, in which viral replicative RNA and virus particles localize close to lipid droplets (14, 15). Moreover, the knockdown of DGAT1/2 in a single infectious cycle decreases the extracellular, but not the intracellular SARS-CoV-2 load, while it reduces the viral nucleoprotein (NP) production (16). Xanthohumol also inhibits coronaviruses protease Mpro in enzymatic assays impairing infection of SARS-CoV-2 and PEDV *in vitro* (17).

Both NP and Spike associate with ADRP, a lipid droplet structural protein, in lung cells of SARS-CoV-2-infected hamsters. Moreover, silencing ADRP suppressed the SARS-CoV-2 load (16). Whereas the results of Dias and coworkers corroborate the idea that lipid droplets serve as platforms for virus replication (190–192), the study of Yuan and coworkers (2021) suggests they participate in a post-replication effect. The ectopic transfection of NP in Huh7 cells, but not of other viral proteins, activates DGAT-1/2 expression (16). This result contrasts with the study of Wang and collaborators (2023) that shows that among the SARS-CoV-2 proteins (except nsp3), only ORF3a induces lipid droplet accumulation in HeLa cells. The authors also show that ORF3a is necessary for lipid droplet accumulation and virus replication in Caco-2 cells using a trans-complementation system (18). Interestingly, ORF3a also shows other activities that favor SARS-CoV-2 infection, such as inhibition of autophagy (193–195), lysosomal egress (196) and IFN signaling (197). Infection of Vero E6 cells with SARS-CoV-2 resulted in the accumulation of lipid droplets, often connected to mitochondria. In contrast, SARS-CoV-infected Vero E6 cells do not present lipid droplets. Type II pneumocytes of COVID-19 patients also exhibit a large amount of lipid droplets and connections between these organelles and mitochondria (198).

Avasimibe, an inhibitor of ACAT, reduces the production of esterified cholesterol and, consequently, lipid droplets (110). It also augments the free cholesterol content of the plasma membrane in Vero E6 cells, in line with a redistribution of cholesterol from lipid droplets to the plasma membrane. Besides the inhibitory effect on SARS-CoV-2 attachment, avasimibe also reduces the replication of SARS-CoV-2 RNA. Interestingly, supporting evidence for the role of ACAT during SARS-CoV-2 infection comes from studies using a genetic approach of loss- and gain-of-function that indicated ACAT2 plays a preponderant part in inhibiting SARS-CoV-2 infection compared to ACAT1 (152).

Here, it is worth reminding that using exogenous 25HC inhibits coronavirus infection, despite the increase in lipid droplet formation. In this case, the effect of 25CH is attributed to the depletion of the accessible pool of cholesterol in the plasma membrane, affecting virus entry by fusion, which is a preceding event relative to the replication phase, where lipid droplets are necessary. Thus, although coronavirus infection induces *CH25H*, endogenous 25CH cannot hamper infection effectively as it happens with the addition of exogenous 25CH (19).

The occurrence of lipid droplets in a cell depends on their biogenesis and lipolysis. Viral infection provokes an increase in lipid droplets, followed by the disappearance of these organelles and a concurrent rise of intracellular FFA and glycerol. While the production of lipid droplets favors virus replication in the first hours of infection, lipolysis benefits virus replication and the production of pro-inflammatory cytokines at later time points. Atglistatin and CAY10499 are selective and non-selective lipase inhibitors, respectively. When given at 12 or 18 hpi of SARS-CoV-2 infected-Vero E6 cells, these drugs prevent the lipid droplets’ lipolysis and rising of FFA and glycerol levels. Notably, the reduction of FFA is associated with decreased fatty acid oxidation and Spike palmitoylation, contributing to the inhibition of virus replication and production of TNF-α, IL-6, and MCP-1. Supplementation of FFA reverts the inhibition of virus replication. Thus, lipid droplets are not just platforms for virus replication but are lipid sources for energetic demands and protein modification during viral infection. CAY10499 and Atglistatin also effectively suppress SARS-CoV-2 infection and the associated inflammatory response *in vivo*, as described in session 5 of this review (199). Interestingly, FFA binds Spike, rendering it unavailable for interaction with ACE2, diminishing SARS-CoV-2 infection (200). The contrasting effects of FFA may be related to the time course of infection.

### 2.6 Membrane remodeling and autophagy during coronavirus infection

Coronavirus infections induce extensive membrane remodeling in the host cell, creating double-membrane vesicles (DMVs) and convoluted membranes (CMs), which shelter replication-transcription complexes (RTCs). These complexes harbor viral proteins and host factors, maintaining a lipid microenvironment that protects newly synthesized RNA molecules from nucleases and innate immune sensors (201). The viral non-structural proteins nsp3, nsp4, and nsp6 are involved in the biogenesis of DMVs (the replication platforms of coronaviruses), which are connected and probably originate from the ER (202–207). The ectopic expression of nsp3 and nsp4 induces the formation of DMVs (203, 205, 208, 209), while the nsp6 bridges DMVs to the ER and to lipid droplets, allowing lipids, but not proteins, to flow from the ER to the DMVs (123). The nsp6 of alpha- and beta-coronaviruses also induces the formation of autophagosomes decorated with LC3 in an ATG5- and PI3K-dependent manner (210). Accordingly, treatment with K22, a drug that targets nsp6, inhibits infection of several coronaviruses (123, 211).

DMVs are like the autophagosomes, regarding their double-membrane constitution and the presence of LC3-I, and they emerge from the ER through mechanisms that involve host autophagic and the ERAD machinery (212, 213). The role of autophagy in coronavirus infection is controversial, though, and each coronavirus exploits the autophagic machinery in a particular way (214). Prentice and coworkers (2004) showed that deletion of the *ATG5* gene inhibits the replication of murine hepatitis virus (MHV) strain A59 in stem cells (215). However, Zhao and collaborators (2007) reported that *ATG5* is dispensable for efficiently replicating the same virus strain in murine bone marrow-derived macrophages or primary murine fibroblasts (216). Thenceforth, extensive literature shows that coronaviruses explore the autophagic process to produce the membrane system that allows its replication while hampering the completion of autophagy, avoiding its degradation in autophagosomes (193–195, 209, 217–222).

During autophagy, autophagosome membranes expand drastically, a process dependent on cellular factors, as will be briefly described. In nutrient-rich conditions, mTOR, a master suppressor of autophagy, interacts with and inhibits the ULK1 complex formed by ULK1, ATG13, FIP200, and ATG101. Stressing conditions relieve mTOR inhibition, and ULK1 translocates to the ER, recruiting the PI3K complex II (PI3KC3-C2), formed by VPS34, Beclin1-1, VPS15, and ATG14. PIP3KC3 originates from PIP3-rich domains in the ER, where the phagophore formation initiates. Assembly of PI3K3 complex depends on the recruitment of WD repeat domain phosphoinositide interacting 2b (WIPI2B) and double FYVE Containing Protein (DFCP1). WIPI2B binds and engages ATG12, ATG5, and ATG16L1 that lipidates microtubule-associated protein light chain 3 (LC3) and γ-aminobutyric acid receptor-associated proteins (GABARAPs) to membrane PE, originating LC3II, a signature of the autophagic process. LC3 II then regulates the elongation and closure of the autophagosomes carrying the material to be degraded after fusion with lysosomes (reviewed by Nakatogawa, 2020) (223).

Autophagosome formation also requires bulk lipid transfer from the ER’s cytosolic leaflet to the phagophore’s cytosolic leaflet. This process is associated with lipid movement between the cytosolic and the luminal leaflets of each organelle to rebalance the amount of lipids between leaflets. These tasks are executed by the ATG2 lipid transfer protein and the scramblases VMP1 and TMEM41B, respectively (224–228). VMP1 also regulates the contact sites of the ER with the phagophore and other organelles, such as mitochondria, endosomes, and lipid droplets (229). Similar to their role in the expansion of autophagosomes, they are also crucial for the formation of DMVs during coronavirus MHV-A59 infection or after ectopic expression of SARS-CoV-2 nsp3 and nsp4 non-structural proteins (230). VMP1 and TMEM41B were identified as important host factors for virus infection in genome-wide screens (231, 232), and genetic analysis revealed that deficiency of each of these proteins blocks DMV formation in distinct ways. In VMP1 KO cells, nsp3/4-bound DMVs are unable to close, while in TMEM41B KO cells, nsp3/4 complexes are unable to form. Interestingly, both proteins function as cholesterol, and PS scramblases (230), but the inhibition of PS synthesis partially rescues the defect of DMV production only in the absence of VMP1 (233). Deficiency in TMEM41B and VPM1 functions leads to the accumulation of accessible cholesterol in the cytosolic leaflet of the plasma membrane (227) and results in the formation of enlarged lipid droplets. In TMEM41B KO cells, this is due to a decrease in the mobilization of fatty acids from lipid droplets to mitochondria and their ß-oxidation (224). The absence of VMP1 also induces large lipid droplets because of the increased lipid flux from the ER to lipid droplets (229).

A genome-wide CRISPR screen of human coronaviruses-infected cells identified genes of the phosphatidylinositol phosphate biosynthesis and the cholesterol homeostasis host pathways, indicating the importance of lipid metabolism in coronavirus infection. Many of these genes were further validated by examining the effectiveness of virus infection in loss-of-function experiments, in which susceptible cells were manipulated to harbor mutant candidate genes. Moreover, several drugs targeting lipid metabolism exhibited inhibitory activity on viral replication: SAR405 (a selective and ATP-competitive inhibitor of class III PI3K - PIK3C3), YM201636 (a PIKfyve inhibitor), PF-429242 (a reversible, competitive aminopyrrolidineamide inhibitor of MBTPS1), 25HC (which promotes ER retention of the SCAP/SREBP complex), Fatostatin (which binds to SCAP and inhibits ER-to-Golgi translocation of SREBPs), Bardoxolone (an activator of the KEAP1-NRF2 complex) (232). Notably, apilimod (a PIKfyve inhibitor) also presents antiviral activity (234–236).

Class III PI3K (also known as VPS34), an essential player in the formation of autophagosomes and vesicular sorting and trafficking, is also central to the formation of DMVs. The VPS34 inhibitors, VPS34-IN1, PIK-III, and SAR405, all inhibit SARS-CoV-2 replication in Calu-3 cells. These drugs may act during early or later steps of the viral infection (14). Other inhibitors of fatty acid metabolism show antiviral activity in post-entry steps: orlistat (inhibits gastric lipases and FASN); triacsin C (targets long chain ACS); TOFA (targets acetyl-CoA) and C75 (inhibits FASN) (14).

Interestingly, both etomoxir (an inhibitor of carnitine palmitoyltransferase 1A -CPT1A), which blocks translocation of fatty acids into the mitochondria, and trimetazidine (inhibits long-chain 3-ketoacyl-CoA thiolase) do not impair SARS-CoV-2 infection, ruling out a role of β-oxidation in viral replication. Furthermore, the inhibitors VPS34-IN1, orlistat, TOFA, or A922500 inhibit the formation of replicative centers based on dsRNA labeling. Furthermore, TOFA and A922500 show much higher inhibition when the viral titers were analyzed, suggesting they have other additional effects besides inhibiting RNA synthesis (14).

The deletion of FASN in Caco-2 cells confirmed the central role of fatty acid metabolism in viral replication, while supplementation with palmitate and oleic acid was partially able to reverse FASN deficiency (14). Yuen and coworkers (2021) also reported an inhibitory effect of VPS34-IN1 in the replication and infectivity of SARS-CoV-2 in VeroE6 cells. VPS34-IN1 and its analog, compound 19, also impair viral infection of *ex-vivo* human lung tissue (221). They also analyzed the effects of other autophagy inhibitors (targets in parentheses) during viral infection of VeroE6 cells. They found that SBI-0206965 (ULK1), 3-methyladenine – 3-MA (class I PI3K), and hydroxychloroquine – HCQ (acidification of autophagolysosome) impaired viral replication. Interestingly, inhibition of ULK1, which is upstream of VPS34 in the autophagy pathway, stimulated SARS-CoV-2 infection instead of inhibiting it. Moreover, 3-MA showed a minimal inhibitory effect, while HCQ strongly inhibited SARS-CoV-2 replication. Twu and collaborators (2021) also report a central role of the class III PI3K in SARS-CoV-2 replication. The VPS34 inhibitor, VPS34-IN-2 (also known as PIK-III), inhibits nsp3/4-induced DMV formation and SARS-CoV-2 replication in A549/ACE2 and Calu-3 cells. Knockdown of VPS34 and Beclin 1 or the PI3P effector DFCP1 cells have the same effect in the A549 cell model (209). Taken together, these data reinforce the importance of lipid metabolism for SARS-CoV-2 infection and its potential as a therapeutic target.

## 3 Therapeutic targeting of the cellular lipid metabolism against coronavirus infection – *in vivo* studies

The dependence of the coronaviruses cycle on host lipids and lipid metabolism motivated the search and assessment of potential antiviral drugs targeting these pathways. In this section, we will focus on drug testing data obtained *in vivo* using COVID-19 animal models and human clinical trials.

Statins are a class of drugs that target hydroxymethyl-glutaryl Coenzyme A (HMG-CoA) reductase, the rate-limiting enzyme in cholesterol biosynthesis. They are widely used in cardiovascular patients, and since cardiovascular disease is a risk factor for developing severe COVID-19, several studies were conducted to evaluate the effect of statins on COVID-19 patients. Besides their cardiovascular effects, statins protect against SARS-CoV-2 infection and inflammatory response triggered by the virus, both *in vitro* and *in vivo* (7, 237, 238). Teixeira and coworkers demonstrated that simvastatin reduces viral replication and lung damage and delays the mortality of SARS-CoV-2-infected K18-hACE2 mice. To our knowledge, this is the only study showing the effectiveness of a statin in a COVID-19 animal model. *In vitro*, simvastatin effectively reduced the binding and internalization of SARS-CoV-2 due to the dislodgement of ACE2 from lipid nanodomains (7). Other studies using *in vitro* settings also indicate the role of statins in constraining SARS-CoV-2 infection (237, 238). Besides ACE2 localization in lipid nanodomains, statins may have other effects due to the broad participation of cholesterol in several steps of the viral cycle. For example, statins inhibit the production of lipid droplets (239), viral assembly (240), and protein post-translational modification and trafficking (241, 242). In addition, statins have anti-inflammatory, immunomodulatory, and antithrombotic effects, including during coronavirus infection (7, 241, 243–246). Indeed, numerous studies indicate that the use of statins is associated with reduced admission to the intensive care unit, faster recovery, and lower risk of mortality among patients on statin medication (247–252). However, some studies suggest that statins do not improve COVID-19 outcomes (253) and can even increase the risk of severe COVID-19 development (254). Meta-analysis of retrospective studies shows controversial results about the beneficial effect of statins in reducing the risk of mortality due to COVID-19 (255–260).

Intraperitoneal injection of the retinoid derivative AM580, shown to modulate cholesterol metabolism and SARS-CoV-2 infection *in vitro*, also rescued body weight loss and death of MERS-CoV-infected hDPP4-transgenic mice. The drug reduced virus load, alveolar damage, and lung inflammatory infiltration (13). Other drugs that affect not only the synthesis but also the traffic of cholesterol through the endosomal pathway, such as itraconazole, fluoxetine, and U18666A, were also tested as adjuvant therapy against coronavirus infection *in vitro* and *in vivo* (261–265). Itraconazole - an ergosterol biosynthesis inhibitor and cholesterol traffic inhibitor - showed antiviral effects *in vitro* against feline coronavirus (FCoV) and SARS-CoV-2 (263, 264). However, it did not suppress the SARS-CoV-2 load in the golden hamster model, which determined the interruption of a clinical study with COVID-19 patients (263, 266, 267). U1866A - an inhibitor of oxidosqualene cyclase, desmosterol reductase, and Niemann-Pick type C1 (NPC1) cholesterol transporter - suppresses intracellular cholesterol biosynthesis and trafficking of cholesterol from lysosomes. In cats, this drug inhibited FCoV replication *in vitro* in an NPC1-dependent manner (266), but *in vivo*, testing was inconclusive due to the small number of animals used (265).

The cholesterol metabolism also generates oxysterols, which modulate the immune and inflammatory response. The effects of 25HC have been described in a previous section of this review and will be summarized here. Several studies report the antiviral and anti-inflammatory activities of 25HC against SARS-CoV-2 infection in animal models (19, 24, 27, 28). Some also propose combinations of 25HC with other molecules to improve its delivery to the lungs (27) or enhance its antiviral activity (268). However, there is a divergent study reporting no protective effects of 25HC against infection, and even a possible potentiation of the pro-inflammatory response induced by 25HC (29), as described in section 2.3.

The antidepressant fluoxetine, and other representatives of this class of drugs, modulate the sphingolipid metabolism and their effects against SARS-CoV-2, using animal models or treatment of COVID-19 patients, were promising. In this context, fluoxetine suppresses SARS-CoV-2 replication in K18-hACE2 mice and the production of pro-inflammatory cytokines and chemokines, like IL-6, TNF-α, CCL2, and CXCL10. Differently from the results obtained in *in vitro* studies, fluoxetine did not alter ceramide and sphingomyelin levels in the lungs but enhanced the levels of hexosylceramide (glucosylceramide) and the ratio HexCer/Cer (41). These effects are encouraging since the metabolomics analysis of COVID-19 patients with pneumonia show increased ceramide levels and decreased long HexCer levels, both associated with disease severity (269). Nasal epithelial cells from healthy volunteers pre-treated with Amitryptiline were resistant to *ex-vivo* infection by SARS-CoV-2 (40). Moreover, Fluvoxamine reduced clinical signs of severe COVID-19 patients compared to placebo-treated controls (270).

Another study comparing the effects of various antidepressants, including SSRIs and tricyclic ones, in hospitalized COVID-19 patients, reported a lower risk of intubation and death among patients that began the use of antidepressants in the first 48h of hospital admission compared to patients who did not take the drugs (271). Home use of antidepressants is also associated with decreased hospitalization of COVID-19-positive patients (272).

Regarding fatty acid metabolism, orlistat suppressed SARS-CoV-2 infection in two mouse models: K18-hACE2 mice and adenovirus-hACE2 C57BL/6J mice. Orlistat improved survival rates and decreased the viral load and lung inflammation when used simultaneously with the virus inoculation. However, when given the day after infection, it delayed the course of the disease but was unable to prevent death (178).

Pharmacological inhibition of enzymes involved in lipid droplet metabolism showed promising results as therapeutic strategies to fight SARS-CoV-2 infection and the associated inflammatory response in the golden-hamster model (16, 152, 199). The use of Xanthohumol – a DGAT-1/2 inhibitor – or atglistatin (inhibitor of LD-associated lipases) diminished SARS-CoV-2 replication and inhibited the production of IFN-γ, IL-6, and TNF-α. The former also reduced inflammatory infiltration and consolidation of the lung parenchyma in a golden hamster model (16, 199). The combination of atglistatin and remdesivir showed synergistic effects reducing the lung lesions provoked by three different SARS-CoV-2 strains.

## 4 Discussion

The success of coronavirus infection relies on the usage of cell lipids. Coronaviruses dock at the plasma membrane and invade cells either by fusing their envelope directly to the cell membrane or by endocytosis mediated by distinct pathways, including lipid nanodomains/caveolae. After entry, they reach the cytosol and induce the synthesis and extensive remodeling of internal membranes that form the replicative organelles of coronaviruses (273). Besides, coronaviruses elicit the formation of lipid droplets, which serve as replication platforms and an energy source (15, 123). Lipid droplets also contribute to the production of inflammatory mediators induced by the infection (15). In order to support a successful infection, coronaviruses prevent the completion of the autophagic process (193–195, 274), hampering the maturation of the autophagosome and use the exocytic route to exit the cell (218).

**Figure 3:**
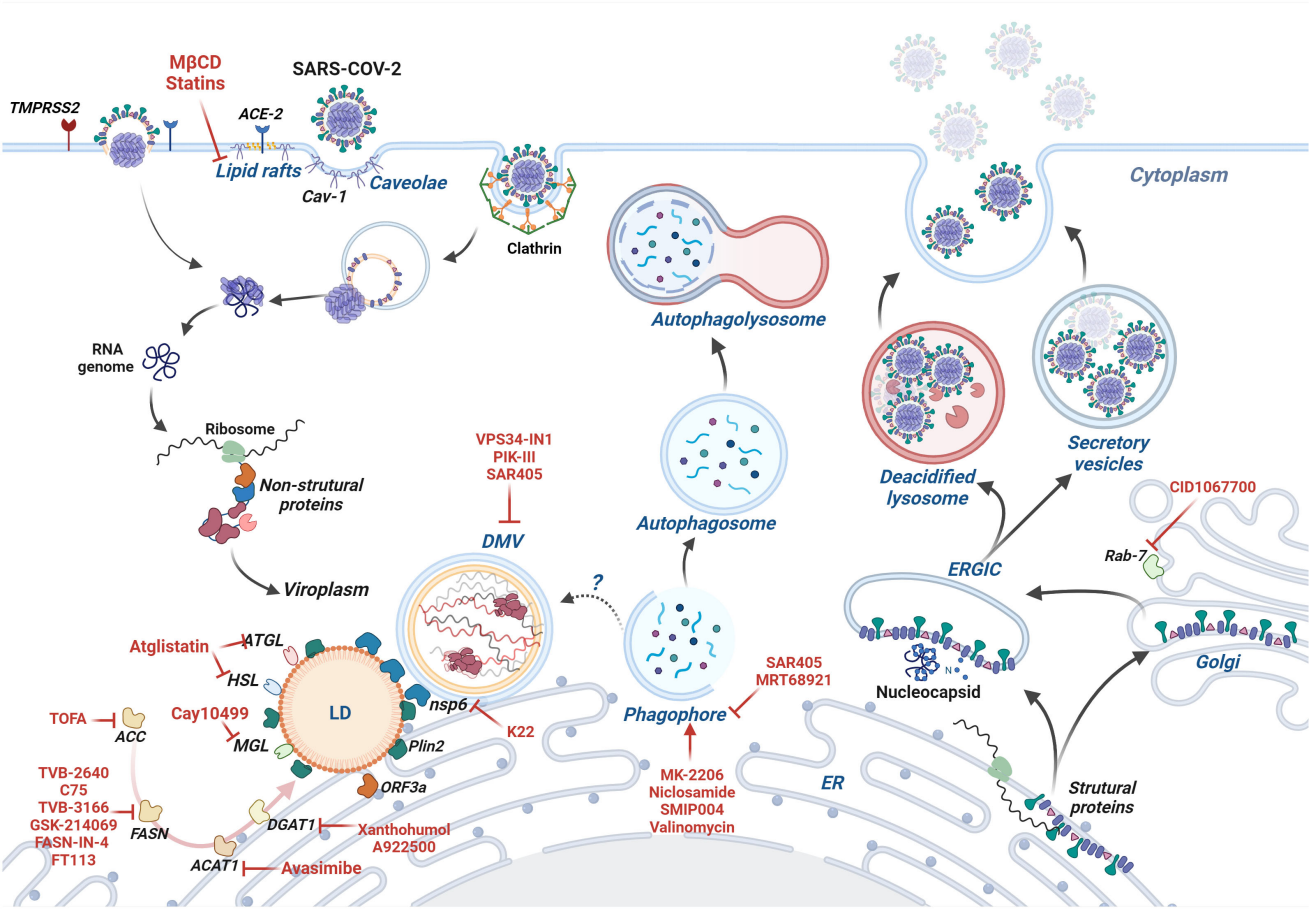
The coronavirus infection and cell lipid metabolism - SARS-CoV-2 cycle is depicted as a model of the coronavirus cycle. SARS-CoV-2 enters the cell by direct fusion with the plasma membrane or clathrin-mediated endocytosis. Caveolae-dependent-endocytosis is not involved in SARS-CoV-2 but is used by other coronaviruses. Once in the cell, the viral genome is transcribed, and non-structural proteins, such as nsp3 and nsp4, induce the formation of double-membrane vesicles, which provide a protected niche for genome replication. Nsp6 bridges DMVs to the ER and lipid droplets, allowing lipids to flow to DMVs. ORF3a, a viral accessory protein, induces the formation of lipid droplets that serve as platforms for virus replication and post-replication events. Drugs affecting cholesterol and fatty acid metabolism inhibit the formation of lipid droplets and viral infection. DMVs share many characteristics with autophagosomes, and there is controversy about whether coronaviruses sequester the autophagic machinery to induce DMVs. Late steps of autophagy are blocked by coronavirus proteins, such as ORF3a, hampering the formation of autolysosomes and virus degradation. The virus particles are assembled during their traffic through the ER, ERGIC, and Golgi until they leave the cell. The egress involves deacidified lysosomes, recycling endosomes and other virus-containing vesicles from the secretory pathway. Drugs targeting molecules of lipid metabolism are written in red, and their mode of action is described in the review and summarized in **Table 2**. Image created using BioRender.

In conclusion, coronaviruses can modulate the cell lipid metabolism in many aspects that favor viral infection and spread. Hence, a broad spectrum of drugs targeting lipid metabolism inhibits coronavirus infection and the subsequent inflammatory and immune responses (**Figure 3** and **Table 2**). Among the drugs described here, some are already FDA-approved; others still require safety and efficacy tests before being released for human treatment. Drugs targeting the host instead of virus factors may be a preferable strategy to prevent the selection of drug-resistance variants (277). Additionally, treatments combining drugs (25, 262, 278) aimed at distinct targets are also a promising therapeutic strategy since this reduces the odds of drug resistance variants and allows the use of lower drug doses. On the other hand, some drug combinations are harmful, such as the combined use of simvastatin and Paxlovid, as the latter inhibits simvastatin metabolization, increasing the risk of cardiovascular problems among statin users. In this case, simvastatin can be substituted by fluvastatin or pravastatin, which can be safely used with Paxlovid (279, 280). A *in silico* study indicated that fluvastatin binds the SARS-CoV-2 M^pro^ protein, which is the target of Paxlovid. Thus, the combined use of these drugs may potentially provide a dual inhibition of M^pro^, which may be particularly relevant since Paxlovid is used for just five days, and infection rebounds in some patients after stopping the use of this drug (280). Another aspect of the pharmacology against coronaviruses that should be appreciated is the discovery that SARS-CoV-2 infection is affected by circadian regulators, such as Bmal1 and CIART (180, 281), the latter acting on the fatty acid metabolism. So far, drug therapy against coronaviruses has not considered this information (282, 283).

**Table 2:** Effects of modulation of lipid metabolism on coronavirus infection.

Drugs/inhibitors	Target	Effects	References
2-bromopalmitate	PAT	Inhibits SARS-CoV-2 Spike palmitoylation in HEK293 cells	(33, 182)
		Inhibits entry of SARS-CoV-2 pseudovirus infection in HEK293T cells	(189)
		Inhibits Spike-mediated cell fusion in HEK293T cells	(33)
		Reduces infection of SARS-CoV-2 in HEK293T cells and Calu-3 cells	(14, 33)
3-MA	VPS34/PIK3C3	Minimal inhibitory effect on SARS-CoV-2 infection in Vero E6 cells	(221)
4-HPR	DES1	Inhibits cell fusogenicity (in a DES-1 independent manner)	(45)
		Inhibits SARS-CoV-2 infection in Vero E6-TMPRSS2 cells	
25HC	Cholesterol metabolism	Inhibits entry of PEDV in Vero cells, of TGEV in ST cells, and PDCoV in LCC-PK and IPI-FX cells	(21–23)
		Inhibits infection of hCoV-OC43 in Huh7.5 and Huh7.5.1 cells and of hCoV-229E in MRC-5 cells and Huh7.5.1 cells	(20, 29, 232)
		Inhibits fusion/entry of SARS-CoV, MERS-CoV pseudovirus in Calu-3 and of SARS-CoV-2 pseudovirus in Calu-3, Caco-2, HEK293-hACE2, Huh7 cells and Vero cells	(19, 24, 28)
		Inhibits SARS-CoV-2 infection in Vero E6 and Huh7.5.1 cells	(25, 28, 232)
		It does not inhibit SARS-CoV-2 infection in TMPRSS2-expressing VeroE6 cells	(29)
		Inhibits SARS-CoV-2 infection in hACE2 mice	(28)
		Inhibits SREBP-activated inflammatory cytokine/chemokine production in PBMC of severe COVID-19 patients	(27)
		Stimulates inflammatory cytokine/chemokine production in SARS-CoV-2-infected K18-hACE2 mice	(29)
A922500	DGAT1	Inhibits SARS-CoV-2 infection in human primary monocytes and Vero E6 cells	(14, 15, 123)
		Inhibits cell death and inflammatory cytokine/chemokine production in SARS-CoV-2 infected monocytes	(15)
AKS466	aCDase	Inhibits SARS-CoV-2 infection in Huh.7, Vero and Calu-3 cells	(42)
AM580	Lipogenesis- and cholesterol-metabolism-regulating gene expression	Inhibits MERS-CoV infection *in vitro* (Calu-3 cells) and *in vivo* (hDPP4-transgenic mice)	(13, 25)
		Inhibits SARS-CoV-2 infection in VeroE6 cells	(261)
Amiodarone	ASM, cholesterol metabolism	Inhibits SARS-CoV-2 infection in Calu-3 cells	(40)
Amitriptyline	ASM	Inhibits SARS-CoV-2 pseudovirus infection in Vero E6 and *ex vivo* in freshly isolated nasal epithelial cells	(234, 236)
Apilimod	PIKfyve	Inhibits SARS-CoV-2 infection in Vero E6 and A549-ACE2 cells	(235)
		Inhibition of entry of SARS-CoV-2, SARS-CoV in 293/hACE2 cells, MERS-CoV pseudovirus in HeLa/hDPP4 cells, and MHV pseudovirus in HeLa/mCEACAM cells	(23)
Asiaticoside	TGF-β	Inhibits PDCoV infection in LLC-PK1 cells	(23)
Atglistatin	ATGL, HSL	Inhibits palmitoylation of SARS-CoV-2 Spike in Vero E6 cells	(238)
		Inhibits SARS-CoV-2 infection in Vero E6 cells	
		Inhibits inflammatory cytokine/chemokine production of SARS-CoV-2 infected Vero E6 cells	
		Inhibits SARS-CoV-2 infection in Syrian hamsters	
		Inhibits inflammatory cytokine/chemokine production, lung injury and mortality in Syrian hamsters	
Atorvastatin	HMG-CoA reductase, SARS-COV-2 RdRp?, SARS-CoV-2 3CL?	Inhibits SARS-CoV-2 infection in Vero E6 cells	(152)
Avasimibe	ACAT	Disrupts the association of ACE2 and in GM1 lipid rafts, perturbing viral attachment	(152)
		Inhibits SARS-CoV-2 pseudovirus infection in VeroE6, VeroE6-TMPRSS2, and Calu-3 cells	
		Inhibits SARS-CoV-2 infection in Calu-3 cells	
		Inhibits SARS-CoV-2 RNA replication in TMPRSS2-VeroE6 cells	
		Expands acutely activated SARS-CoV-2-specific T cells, without affecting memory or non-activated T cells	
Bardoxolone	KEAP1-NRF2 complex	Inhibits SARS-CoV-2, hCoV-OC43 and hCoV-229E infection in Huh7.5.1 cells	(232)
Bis-piperazine backbone-based compounds	DHHC9	Inhibits palmitoylation of SARS-CoV-2 Spike in Caco-2 cells	(189)
		It does not inhibit entry, assembly or egress of SARS-CoV-2 pseudovirus in HEK293T cells	
		Inhibits SARS-CoV-2 pseudovirus infection and fusion of HEK293T cells	
		Inhibits SARS-CoV-2 infection and fusion of Caco-2 cells	
C75	FAS	Inhibits SARS-CoV-2 infection in Huh 7.5, Vero-E6, and Hela-ACE-2 cells	(179)
CAY10499	MGL, HSL, FAAH	Inhibits palmitoylation of SARS-CoV-2 Spike in Vero E6 cells	(199)
		Inhibits SARS-CoV-2 infection in Vero E6 cells	
		Inhibits inflammatory cytokine production in SARS-CoV-2-infected Vero E6 cells	
Ceranib-2	aCDase	Inhibits SARS-CoV-2 infection in Huh7 cells	(42)
Cerulenin	FAS	Inhibits SARS-CoV-2 infection in Huh 7.5, Vero-E6, and Hela-ACE-2 cells	(179)
CID1067700	RAB7	Inhibits MHV egress	(218)
EGCG	FAS	Inhibits SARS-CoV-2 infection in Huh 7.5, Vero-E6, and Hela-ACE-2 cells	(179)
Etomoxir	CPT1A	Does not inhibit SARS-CoV-2 infection in Calu-3 cells	(14)
FASN-IN-4	FAS	Inhibits SARS-CoV-2 infection in HEK293T-hACE2 cells	(178)
Fatostatin A	SCAP	Moderately inhibits SARS-CoV-2 infection in Huh7.5.1 cells	(232)
		Inhibits activation of SREBP2 and inflammatory cytokine production in PBMC of severe COVID-19 patients	(12)
Fluoxetine	ASM, aCDase	Inhibits SARS-CoV-2 infection in Vero E6, Calu-3, polarized Calu-3 cells and in human lung tissue slices	(170, 261)
		Inhibits SARS-CoV-2 infection in human A549 lung carcinoma cells and 2D human airway cells	(41)
		Inhibits SARS-CoV-2 infection in K18-hACE2 mice	
		Inhibits inflammatory cytokine production in SARS-CoV-2-infected Vero E6 cells	
Fluvoxamine	HMG-CoA reductase	Reduces clinical signs of severe COVID-19 patients	(270)
Fluvastatin		Inhibits hCoV-2297 and SARS-CoV-2 infection in Huh7.5 and Calu-3 cells, respectively	(237)
		Moderate inhibition of SARS-CoV-2 infection in air-liquid interface cultures of HBEC cells	
		Reduces the expression of seven SARS-CoV-2 proteins, mainly ORF1ab, ORF3, and ORF6	
		Does not downregulate the expression of TNF-modulated proteins in SARS-CoV-2 infected cells in contrast to simvastatin and rosuvastatin	
FT113	FAS	Inhibits SARS-CoV-2 infection in HEK293T-hACE2 cells	(178)
Genz-123346 and Genz-667161	GCS	Inhibits the early steps of SARS-CoV-2 replication in Vero E6 cells	(43, 44)
GSK-214069	FAS	Inhibits SARS-CoV-2 infection in HEK293T-hACE2 cells	(178)
Imipramine	Cholesterol and sphingolipid metabolism,	Inhibits SARS-CoV-2 infection in Calu-3 cells	(261)
Itracononazole	NPC1, cholesterol metabolism,	Inhibits SARS-CoV-2 infection in Calu-3 cells, Vero E6 and Caco-2 cells	(262, 263)
		Inhibits FCoV infection in fcwf-4 cells	(275)
		Does not reduce SARS-CoV-2 infection in golden hamsters	(267)
K22	nsp6	Inhibits infection of several coronaviruses (FCoV-RL in FCWF cells, MHV-Gluc in L-929 cells, IBV and SARS-CoV in Vero cells, hCoV-2297 and MERS-CoV in HAE cells)	(211)
		Inhibits lipid flowing from the ER and lipid droplets to DMVs and inhibits infection of SARS-CoV-2 in Calu-3 cells	(123)
MβCD	Plasma membrane cholesterol	Inhibits MHV infection and cell-cell fusion in HeLa-CEACAM and DBT cells (dislodges CEACAM from lipid rafts to non-raft domains)	(1, 153)
		Inhibits hCoV-229E entry/infection in Huh7.5 cells in primary human fibroblasts	(2)
		Inhibits hCoV-OC43 infection in HCT-8 cells	(10)
		Inhibits TGEV infection in ST cells	(164, 165)
		Inhibits SARS-CoV pseudovirus in Vero CCL-81 cells and SARS-CoV infection in Vero E6 cells (dislodges ACE2 from rafts to non-raft domains)	(5)
		Inhibits SARS-CoV pseudovirus infection in Vero E6 cells (dislodges ACE2 from lipid rafts to non-rat domains)	(4)
		Inhibits early steps of SARS-CoV-2 infection in Vero E6 cells (reduces expression of ACE2 that does not localize in lipid rafts)	(3)
		Inhibits attachment/infection of IBV in Vero, A549 and DF1 cells (reduces expression and dislodges viral structural proteins from lipid rafts to non-rafts domains)	(6)
Mevastatin	HMG-CoA reductase	Inhibits attachment of IBV in Vero cells (reduces expression of viral structural proteins from rafts to non-rafts domains)	(6)
MK-2206	AKT	Induces autophagy and inhibits SARS-CoV-2 infection in VeroFM cells	(276)
MRT68921	ULK	Inhibits autophagy and promotes SARS-CoV-2 infection SARS-CoV-2 infection in VeroFM cells	(276)
Niclosamide	SKP2	Increases Beclin1 stability, induces autophagy and inhbits SARS-CoV-2 infection in VeroFM cells	(276)
Nystatin		Inhibits hCoV-OC43 infection in HCT-8 cells	(10)
Orlistat	FAS, lipases	Inhibits SARS-CoV-2 infection in Vero E6 cells (post-entry effect), Calu-3 and HEK293T-hACE2 cells	(14, 178)
		Inhibits SARS-CoV-2 infection and lung damage in hACE2-K18 mice	(178)
PF-429242	MBTPS1	Inhibits SARS-CoV-2, hCoV-OC43 and hCoV-229E infection in Huh7.5.1 cells	(232)
PIK-III	VPS34/PIK3C3	Inhibits SARS-CoV-2 infection in Vero E6 and Calu-3 cells	(14)
Rapamycin	mTORC1	Induces autophagy and slightly inhibits SARS-Cov-2 infection in VeroFM cells	(276)
SAR405	VPS34/PIK3C3	Inhibits autophagy and promotes SARS-CoV-2 infection in Calu-3 and VeroFM cells	(14, 276)
		Inhibits SARS-CoV-2, hCoV-OC43 and hCoV-229E infection in Huh7.5.1 cells	(232)
SBI-0206965	ULK1	Does not inhibit SARS-CoV-2 infection in Vero E6 cells	(221)
Simvastatin	HMG-CoA reductase	Inhibits SARS-CoV-2 attachment/entry/infection in Vero E6 and Calu-3 cells (dislodges ACE2 from lipid rafts to non-rat domains)	(7)
		Inhibits inflammatory cytokine/chemokine production in *in vitro* inactivated SARS-CoV-2-infected human neutrophils, human monocytes and Calu-3 cells	
		Inhibits inflammatory cytokine/chemokine production in the lungs of SARS-CoV-2-infected hACE2-K18 mice	
		Inhibits SARS-CoV-2 infection and lung damage in hACE2-K18 mice	
		Do not inhibit SARS-CoV-2 infection in Calu-3 cells	(237)
SMIP004	SKP2	Increases Beclin1 stability and inhibits MERS-CoV and SARS-CoV-2 infection in VeroB4 cells	(222, 276)
SN50	NFκB	Inhibits activation of SREBP2 and inflammatory cytokine production in PBMC of severe COVID-19 patients	(12)
TOFA	ACC	Inhibits SARS-CoV-2 replication/infection in Calu-3 cells	(14)
Triacsin C	ACS	Inhibits SARS-CoV-2 infection in Vero E6 and Calu-3 cells (post-entry effect)	(14)
Trimetazidine	LCKAT	Does not nhibit SARS-CoV-2 infection in Calu-3 cells	(14)
TVB-2640	FAS	Inhibits SARS-CoV-2 infection in HEK293T-hACE2 cells	(178)
TVB-3166	FAS	Inhibits SARS-CoV-2 infection in Huh 7.5, Vero-E6, Hela-ACE-2, and HEK293T-hACE2 cells	(178, 179)
TVB-3664	FAS	Inhibits SARS-CoV-2 infection in HEK293T-hACE2 cells	(178)
U18666A	NPC1	Inhibits FCoV infection in fcwf-4 cells	(261, 275)
		Inhibits SARS-CoV-2 infection in polarized Calu-3 cells	(276)
Valinomycin	SKP2	Increases Beclin1 stability and inhibits SARS-CoV-2 infection in VeroFM cells	(14, 221)
VPS34-IN1	VPS34/PIK3C3	Inhibits SARS-CoV-2 infection in Vero E6 cells and in *ex vivo* human lung tissue culture	(17)
Xanthohumol	DGAT-1/2, coronavirus Mpro	Inhibits Mpro activity in enzymatic assay and inhibits SARS-CoV-2 and PEDV infection in Vero-E6 cells	(16)
		Inhibits SARS-CoV-2 in Calu-3 cells and human embryonic stem cells-derived cardiomyocytes	(16)
		Inhibits SARS-CoV-2 infection in Syrian hamsters	
		Inhibits inflammatory cytokine/chemokine production and lung injury in Syrian hamsters	(232)
YM201636	PIKfyve	Inhibits SARS-CoV-2 infection in Calu-3 and Huh7.5.1 cells	(232)

ACAT (acyl-coenzyme A:cholesterol acyltransferase), ACC (acetyl-CoA carboxylase), ACS (acyl-CoA synthetase), aCDase (acid ceramidase), ASM (acid sphingomyelinase), ATGL (adipose triglyceride lipase), CPT1A (carnitine palmitoyltransferase 1A),DES1 (sphingolipid delta(4)-desaturase), DGAT (Diacylglycerol O-acyltransferase 1), DHCC9 (palmitoyltransferase ZDHHC9), DMV (double membrane vesicles), EGCG (epigallocatechin gallate), FAAH (fatty acid amide hydrolase), FAS fatty acid synthase, GCS (ceramide glucosyltransferase, HAE (human airway epithelial), HMG-CoA-reductase (3-hydroxy-3-methylglutaryl-coenzyme A reductase), HSL (hormone-sensitive lipase), LCKAT (long chain 3-ketoacyl-CoA thiolase), MAGL (monoglyceride lipase), MβCD (methyl-beta-cyclodextrin), NPC1 (Niemann-Pick intracellular cholesterol transporter 1), PAT (palmitoyl acyltransferase), PDCoV (porcine delta coronavirus), PEDV (porcine epidemic diarrhea virus), PIKfyve (1-phosphatidylinositol 3-phosphate 5-kinase), 3CLpro (3CL protease, RdRp (RNA-dependent RNA polymerase), Mpro (viral membrane protein), SARS, CoV (severe acute respiratory syndrome coronavirus), SARS-CoV-2 (severe acute respiratory syndrome coronavirus 2), SREBP (sterol regulatory element binding protein), TGEV (porcine transmissible gastroenteritis virus), TFG-β (transforming growth factor-beta), VPS34/PIK3C3 (phosphatidylinositol 3-kinase catalytic subunit type 3).

## Author contributions

DC-S: Writing – original draft. FP-D: Writing – original draft, Software. AG: Writing – review & editing. CM-M: Writing – review & editing, Writing – original draft. CA: Writing – original draft, Writing – review & editing, Conceptualization, Data curation, Formal Analysis, Supervision.
